# COVID-19 and financial performance: Pre and post effect of COVID-19 on organization performance; A study based on South Asian economy

**DOI:** 10.3389/fpubh.2022.1055406

**Published:** 2023-01-10

**Authors:** Syed Usman Qadri, Zhiqiang Ma, Mohsin Raza, Mingxing Li, Safwan Qadri, Chengang Ye, Haoyang Xie

**Affiliations:** ^1^School of Management, Jiangsu University, Zhenjiang, China; ^2^Department of Management Science, TIMES Institute, Multan, Pakistan; ^3^Department of Public Administration, Wuhan University, Wuhan, China; ^4^Department of Management Science, Business School, University of International Business and Economics, Beijing, China; ^5^School of Information and Computing Sciences, Zhejiang University, Hangzhou, China

**Keywords:** financial performance, pre-COVID-19, post-COVID-19, COVID-19 pandemic, South Asian banking sector, banking performance

## Abstract

The COVID-19 epidemic has damaged developing as well as developed economies and reduced the profitability of several companies. Technological advancement plays a vital role in the company's performance in this current situation. All activities carry on virtually. In this study, the financial performance of enterprises in the South Asian banking industry will be compared before and after the COVID-19 epidemic. Furthermore, the full influence of the pandemic will take place in the long run. This study also explains the technological effect on improving performance, especially during the period of the COVID-19 pandemic. It has an impact on people's social lives as well as the economic world. This study examined a sample of 34 banks from the South Asian region from 2016 to 2021. A Wilcox rank test was used to determine whether there was a significant difference before and after the epidemic era. The overall conclusion of this study is that the COVID-19 pandemic had a significant influence on the bank's financial performance, particularly in terms of profitability. But technological advancement has a positive effect on organizational performance, ultimately increasing the financial performance of South Asian banks. And there is a big difference between pre-pandemic and post-pandemic organizational performance. The findings of this study have significant policy implications since it is clear that cooperation among governments, banks, regulatory agencies, and central banks is necessary to address the financial and economic effects of the COVID-19 pandemic.

## 1. Introduction

The global pandemic COVID-19, a coronavirus pandemic, has had a disproportionate impact on the world's social, economic, political, and religious systems (1). On December 31, 2019, Dr. Tedros Adhanom Ghebreyesus, Director of the World Health Organization, declared the coronavirus a public health emergency (2). The whole corporate world confronts various obstacles in the business climate in 2020, including the collapse of oil prices and the release of a new version of COVID-19 (3, 4). This destructive and upgraded form of COVID-19 not only has an impact on the health and prosperity of the people living in society, but it also causes instability in the global economy (5, 6). After the declaration of WHO COVID-19 as a worldwide pandemic, the whole globe was declared a global lockdown (7). As a result, both corporate and non-profit organizations have been horrified by the international pandemic issue of COVID-19 (3). This widespread shutdown harmed all macroeconomic metrics (e.g., oil prices, unemployment rates, commodity prices, inflation rates, etc.). Ultimately, many organizations' financial performance suffers, and the majority of employees are dismissed or work from home (2). IMF data shows that global GDP will fall by 3.2 percent in 2020. Furthermore, GDP in advanced markets falls by 4.6 percent, GDP in emerging markets falls by 2.1 percent, and GDP in developing markets falls by 2.0 percent. The author estimates that the firms' second quarter revenues in 2020 will decline by 80 percent, 60 percent, and 50 percent, respectively (8). As a result, the development of this pandemic has necessitated tight and prompt measures and policies from local governments as well as local authorities to restrict viral transmission (9). The rapid reaction of the government and local authorities to the spread of this virus led to behavioral and psychological changes in the general public, as well as a reduction in the social gap between them (9). International authorities have also taken rigorous measures such as banning air travel, halting international tourism, restricting ridership, reducing inter-country travel, and so on (9). The spread of the global pandemic affects the east, west, north, and south counties, and the country faces on economic recession (10, 11). Finally, in 2020, the world economy will confront a major threat in the form of the new COVID-19 pandemic. Furthermore, in this pandemic situation, social work has become increasingly difficult to perform due to lockdown (12, 13). Government restrictions are reducing in-person services in the services sector. The service sector includes banks, telecommunications, financial institutions, postal services, insurance companies, and software development firms (14).

Numerous sectors operate in the global economy, but two (manufacturing and services) are regarded as the most important because they contribute significantly to global GDP. According to the IMF's (15), the services sector contributed 63.6 percent of the world's GDP (16). There is a lot of literature available on the manufacturing sector that is affected by the global pandemic. Therefore, I have selected the services sector to learn about the effect of COVID-19. Erden and Aslan (17) concluded that banks are included in the services sector that has been impacted by the pandemic (18). The term “bank” appears for the first time in history in Italy. There is no common definition of a bank in the international literature. A general definition will be made after a few definitions are given here. A bank is defined as an enterprise that accepts deposits from individuals and invests in various sectors in order to pay the individual a margin (19). The financial performance of the banking sector has had ups and downs during COVID-19 (20). The global financial crisis is a major danger to the banking sector in South Asia, as it reduces banking performance (21). A number of other events that occurred in the past will have an impact on the financial performance of the baking sector. The Crimean War of 1854, which took place between Russia and the Ottomans, affected the banking sector. As a result, the Ottoman bank's performance decreased due to that war. The global financial crisis of 2008 also had a great impact on the banking industry in South Asian banks. Ultimately, it will affect the financial performance of the banking sector. Another event in the downfall of tourism in Sri Lanka is that it accounts for 12 percent of the country's GDP (22). It will also affect the banking sector of Sri Lanka As a result, the financial performance of Sri Lanka's banking sector has deteriorated. The financial crisis of 2008 significantly contributed to the lower efficiency of Bangladesh's banking sector. Burki and Niazi (23) discovered that the banking industry's financial performance declined from 1991 to 2000. The author of the study stated that local banks, as well as international banks and Islamic banks, saw a reduction in performance during and post the pandemic period. As a result, financial performance is seen as a tool for analyzing how firms can successfully execute (24).

All of the preceding discussion was about signaling theory because a bad signal is sent to the market in the form of war, a crisis, or pandemic (25). It will decrease the performance of the different companies. The researcher, (26) argues that the market generates both positive and negative signals for business users. As a result, these signals are regarded by business users as information and educate them about the company's present financial health. Positive and negative signals can have an impact on investment decisions and market circumstances. The COVID-19 sends out negative signals to the market and has an impact on every industry of business. People tend to avoid investing after such a terrible epidemic indicator and prefer to cut their losses by withdrawing investments (27). These types of bad signals will also result in a lower level of economic activity and a reduction in economic growth. The existing literature explores several methods to assess a company's financial performance, such as profitability, liquidity, solvency, and activity (24, 28, 29). A lot of methods are available for financial performance, which include the CAMEL Model, VAIC Model, TOPSIS Model, MCDM Model, DEA Model, and the Financial Ratios Analysis Method (30–36).

There has been a significant disruption in the global business world as many firms have been forced to close due to the current COVID-19 epidemic. Based on the above COVID-19 and financial performance, it is required to undertake research to assess the financial performance of the South Asian banking system both before and after the pandemic. But the advancement and development of technology can have a positive impact on employees' performance as well as improve the overall performance of the South Asian banking industry (37). Technological advancement is the combination of creating new knowledge and generating new ideas that will impact the overall performance of companies (38). Internal business progress drives technological advancement, and internal business progress is dependent on the organizational workforce (39). The studies of Alam and Murad (40); Song et al. (41), Sapta et al. (42) explore that there is a close relationship between technological advancement and employee performance. So, advancements in technology, particularly in ICT (information and communication technology), can result in an increase in productivity or enhanced performance (43). Hence, the objective of this research is to forecast financial performance and the effects of COVID-19 on the financial performance of South Asian banks and also to see if there are any variations in financial performance between the pre and post COVID-19 pandemics. Further, how does technological advancement increase the overall performance of the South Asian banks? The contributions of our research are as follows: Theoretically, it multiplies the effects of COVID-19 on the financial performance of South Asian banks across industries. Most of the previous studies carried out have mainly focused on the effects of the COVID pandemic on the manufacturing sector at the macro-level of the economy. Few researchers focused on the effect of the pandemic on South Asian markets or on Pakistan, Sri Lanka, and Bangladesh's financial markets. Practically, it will assist policymakers in developing policies to deal with COVID-19 pandemics or emergency situations. The final part examines the financial impacts of major pandemics and other uncontrollable events on the economy. The reaction of the markets to COVID-19 has already been studied in detail by Liu et al. (44), Narayan et al. (45), and Wang et al. (46).

## 2. Related literature and hypothesis development

### 2.1. Financial performance and ratios analysis

Financial performance analysis is regarded as the most significant analysis since it assists users (investors, shareholders, stakeholders, managers, owners, and so on) in determining whether or not the organization is functioning successfully (47). Ratio Analysis, on the other hand, is a crucial technique for analyzing a company's financial performance (48). It is also beneficial to understand the company's strengths, weaknesses, opportunities, and threats. Based on the information presented above, it is determined that ratio analysis is carried out through the financial statements of the firms in order to learn about the financial performance of the companies. The existing literature (24, 28, 29) divided the ratios into the following main heads, which included profitability, liquidity, solvency, and activity.

#### 2.1.1. Profitability measures

##### 2.1.1.1. Return on assets

There are several ratios that are used to assess a company's earnings and profitability. Balasundaram (49) suggests that ROA is the best way to measure a company's profitability based on these measures. This ratio is used to determine if a company's assets are being used to generate profits as well as how much profit is earned on the basis of the company's assets (50, 51). A higher ratio indicates that the firm is operating well, and vice versa. We use the following formula to compute the ROA: net income/total assets.

H_1_ There is significant difference in return on assets pre and post pandemic of south Asian banks.

##### 2.1.1.2. Earning per share

This ratio is also used to assess the company's profitability. The study of Darya (52) that the EPS demonstrates a company's performance; if the earnings per share are high, the shareholder wealth is maximized, and the company's rate of return is likewise high (53). This ratio is also beneficial to investors, as they must examine it before investing in any stock. Divide net income by the number of shares in circulation to get earnings per share.

H_2_ There is significant difference in earning per share pre and post pandemic of south Asian banks.

#### 2.1.2. Performance measures

##### 2.1.2.1. Return on equity

Different ratios are used to assess corporate performance. ROE is the most important ratio for assessing a company's success. The ultimate goal of an investor is to maximize their wealth and grow the margin on their stock in that firm, which can be measured using ROE (54). The higher the ROE, the greater the shareholder wealth. Divide the company's net income by its total equity to arrive at this ratio.

H_3_ There is significant difference in return on equity pre and post pandemic of south Asian banks.

##### 2.1.2.2. Total assets turnover ratio

Another metric used to assess a company's performance is the Total Assets Turnover Ratio. According to Ellis (55), asset turnover or utilization measures which assets are capable of generating and what the organization really generates from that asset. The research by Jose et al. (56) and Seema et al. (57) demonstrates that asset utilization has a major impact on a firm's financial success. This ratio is calculated by dividing total sales or revenue by total assets.

H_4_ There is significant difference in total assets turnover pre and post pandemic south Asian banks.

#### 2.1.3. Leverage measure

##### 2.1.3.1. Debt to equity ratio

Solvency ratios are another name for leverage ratios. These ratios indicate a company's capacity to satisfy its short-term and long-term obligations through stock or debt (58). A company that has a larger proportion of debt than equity is more likely to fail (59). In the event of insolvency, the corporation has the capacity to pay its debts promptly by liquidating its assets (59). This ratio is calculated by total liabilities, or debt, divided by total equity.

H_5_ There is significant difference in debt-to-equity pre and post pandemic of south Asian banks.

##### 2.1.3.2. Debt to total assets ratio

This ratio, DTAR, is also known as a leverage ratio since it indicates how many assets a firm has to service its debts (5). In other words, it indicates how much debt is covered by the company's total assets. DTAR is used to calculate the amount of leverage, or how much debt is used to acquire assets. This ratio is calculated by dividing total debt by total assets.

H_6_ There is significant difference in debt to assets pre and post pandemic south Asian banks.

## 3. Materials and methods

### 3.1. Study design

This is quantitative research that compares the effects of the pandemic on organizational performance in the banking industry before and after the epidemic. The model is based on earlier research conducted by Daryanto and Rizki (58). The data is collected from the official websites of South Asian banks.

### 3.2. Study model

**Figure 1:**
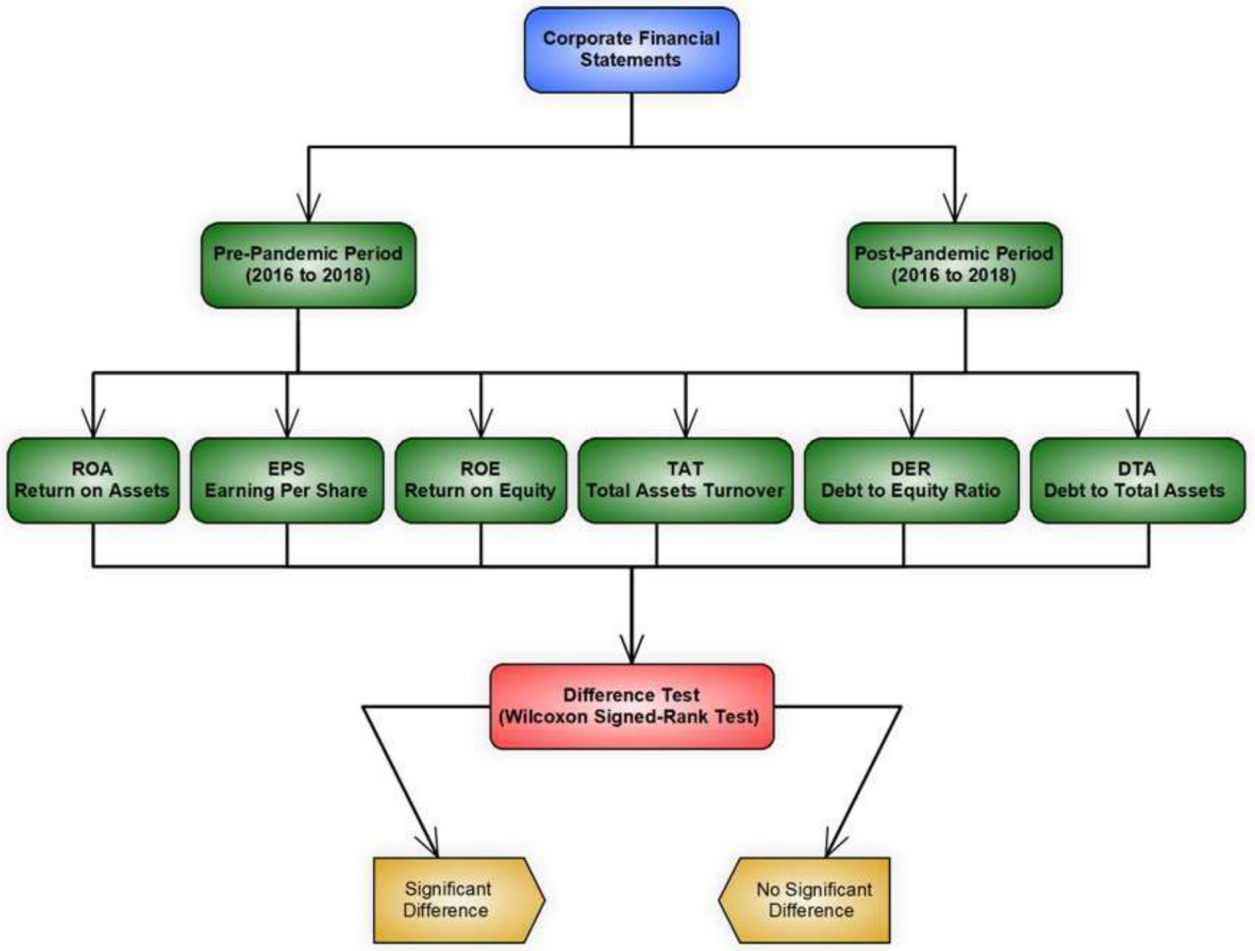
Study model.

Figure 1 represent the model which we have been followed for this study. We have collected the data from official websites of the banks of South Asia. By using the collected data, we find out the ROS, EPS, ROE, TAT, DER and DAT. And also check that there is any difference between the pre and post pandemic period.

### 3.3. Data collection and sources of this study

All of the data used in this study came from secondary sources, including the official websites of South Asian banks. To meet the study's goal, data is gathered for 6 years, with the year chosen depending on the most recent year. All 6 years were separated into two parts: the first 3 years, from 2016 to 2018, were considered pre-pandemic, and the remaining 3 years, from 2019 to 2021, were considered post-pandemic. This study examined a sample of 34 banks from the South Asian region from 2016 to 2021. Only banks with the most recent financial statements from 2021 are included in the data sample; banks without financial statements during that time period are excluded. Then, examine the financial performance of the pre-pandemic and post-pandemic periods. The scope of the study is broad because it includes the banking system of Pakistan, Sri Lanka, and Bangladesh banking systems. With the help of parent articles (58), we determine the population size N for the requirement of an article. Because without knowing the actual population of any study, we can't estimate the data size. In the given reference article, we considered the time period 2016–2020. In this article, we considered period from 2016 to 2021, increasing the time duration due to the improvement of results.

## 4. Results and findings

### 4.1. Correlation matrix

Table 1 shows the correlation between all the studied variables. The result of −1 indicates a perfectly negative linear correlation between two variables, while the result of 0 indicates no linear correlation between two variables, and 1 indicates a perfectly positive linear correlation between two variables. The overall result of the correlation matrix shows that all the variables have a strong positive relationship with each other.

**Table 1:** Correlation matrix of all variables.

	**ROA**	**ROE**	**EPS**	**TATR**	**DER**	**TDTA**
ROA	1					
ROE	−0.22	1				
EPS	0.56	−0.34	1			
TATR	0.02	−0.37	0.29	1		
DER	0.44	−0.24	0.41	0.38	1	
TDTA	−0.39	0.56	−0.52	−0.35	−0.13	1

### 4.2. Financial statistic

**Figure 2:**
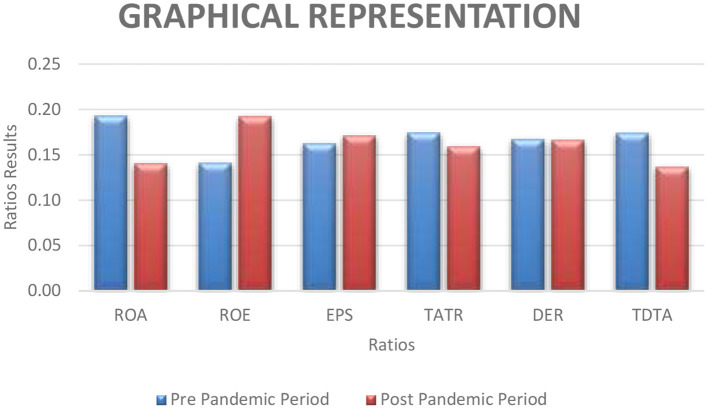
Graphical representation of financial ratios.

The ROA, which gauges the total profitability of the banking industry, has been declining since 2016 and will continue to do so until 2019. Figure 2 also illustrates that the ROA of banks was at its lowest during the post-pandemic period. Figure 2 also reveals that the ROA was 73 prior to the pandemic, but it has fallen while COVID is at its highest in 2019. The EPSPS share ratio is beneficial from the investor's point of view. The EPS in the post-pandemic era is much higher than in the pre-pandemic period. The EPS has now been raised to 6.96 in 2021 because of the banks' profit margin increase in 2021. ROE shows the owner's contribution to the company. The ROE has been improving over the period from 2016 to 2021. In the pandemic period, businesses face losses, and the owner's equity is more equity in their business for survival. The total asset turnover ratio is used to evaluate an organization's performance. The bank's TATR declines significantly from 2016 to 2021, as seen in Figure 2. It was 6.96 before the epidemic, but it has dropped to 6.36 since then. The DER ratio is used to assess a company's leverage; the greater the ratio, the more leveraged and riskier the corporation. The overall results show that the South Asian banks have low leverage. Another ratio used to assess the company's leverage is total debt to total assets. In the epidemic phase, it declines continuously.

Figure 2 represents the overall average of different ratios in the pre- and post-pandemic periods. On the X axis, all the ratios are presented, while on the Y axis, the results of the different ratios are presented. And the graphical representation shows that the results are better in the pre-pandemic period than in the post-pandemic period.

### 4.3. Descriptive statistic

Table 2 displays descriptive statistics for the gathered data, including (minimum value, maximum value, mean, and standard deviation) for all variables of banks listed on the Pakistan Stock Exchange from 2016 to 2021. According to Table 2, the mean value of ROA in the pre-pandemic period was −3.24 and in the post-pandemic period was −1.73. This clearly demonstrates that ROA decreases during the epidemic but increases somewhat in 2021. In the post-pandemic phase, the residual ROE, EPS, TAT, DER, and TDTA all grow. This is because, after the epidemic period is over, the firms' net income rises, which influences all ratios. Based on the findings, we may conclude that the COVID-19 pandemic condition is hazardous for the banking sector of Pakistan.

**Table 2:** Data descriptive statistics results for all variable pre and post pandemic period.

	**N**	**Minimum**	**Maximum**	**Mean**	**Std. deviation**
ROA pre-pandemic	34	0.7238	1.02155	−3.24	2.37
ROA post-pandemic	34	6.6868	7.43101	−1.73	33.73
EPS pre-pandemic	34	0.0685	0.22080	−1.15	0.19
EPS post-pandemic	34	6.2124	3.69723	0.43	14.30
ROE pre-pandemic	34	12.3903	5.43754	−2.13	25.58
ROE post-pandemic	34	1.0300	0.47272	0.84	3.53
TAT pre-pandemic	34	0.5659	1.38359	−5.90	2.13
TAT post-pandemic	34	8.1626	8.30210	−2.43	32.90
DER pre-pandemic	34	0.1294	0.13959	0.02	0.84
DER post-pandemic	34	5.8153	3.08083	0.04	13.19
TDTA pre-pandemic	34	11.8109	7.13973	−13.10	25.49
TDTA post-pandemic	34	0.9618	0.19345	0.85	2.03
Valid N (listwise)	34				

### 4.4. Wilcoxon signed-rank test

The Wilcoxon Signed Ranks Test in Table 3 reveals for the first ROA, the banks have a negative rank of 20 and a positive rank of 13, indicating that the ROA is lower and the Z score is −1.58 shown in Table 4. It indicates that the ROA differs between the before and after pandemic periods. As a result, we may deduce that ROA is falling year after year. As a result, we accept the first hypothesis, which states that there is a difference in return on assets before and after the pandemic, but this difference is not important because it has a.114 meaningful value. In the instance of EPS, it has 10 negative rankings and 24 positives rankings, with a Z score of −2.599 shown in Table 4, indicating that there is a difference in EPS between the pre and post pandemic periods. While the difference is noteworthy since it has a value of 0.001, which is smaller than the value of 0.005. The second hypothesis is likewise accepted on the basis of the first. The next metric is the ROE, which indicates that the banking sector's ROE is higher than it was before the epidemic. As a result, we can infer that the ROE has increased year after year. However, the rank test reveals that it has 15 negative rankings and 13 positive rankings, indicating that the ROE differs between the pre and post pandemic periods. As a result, the third hypothesis, that there is a significant difference in the return on equity before and after the pandemic, is accepted. The performance of banks is judged by the TAT ratio, which has a lower negative rank than a higher positive rank. The Z score for that ratio is −1.957 shown in Table 4, but it is not significant because it is more than 0.005. As a result, the notion that TAT differs between pre and post-pandemic is accepted. Similarly, the DER and TDTA are used to assess bank liquidity. Both have a greater positive rank than a negative rank, and the Z values are −1.510 and −0.644, respectively shown in Table 4, with no significant difference. As a result, the remaining two hypotheses are likewise accepted.

**Table 3:** Wilcoxon signed-rank test results.

		**N**	**Mean rank**	**Sum of ranks**
ROA post-pandemic - ROA pre-pandemic	Negative ranks	20a	18.45	369.00
	Positive ranks	13b	14.77	192.00
	Ties	1c		
	Total	34		
EPS post-pandemic - EPS pre-pandemic	Negative ranks	10d	14.55	145.50
	Positive ranks	24e	18.73	449.50
	Ties	0f		
	Total	34		
ROE post-pandemic - ROE pre-pandemic	Negative ranks	15g	13.57	203.50
	Positive ranks	13h	15.58	202.50
	Ties	6i		
	Total	34		
TAT post-pandemic - TAT pre-pandemic	Negative ranks	22j	17.73	390.00
	Positive ranks	11k	15.55	171.00
	Ties	1l		
	Total	34		
DER post-pandemic - DER pre-pandemic	Negative ranks	11m	17.82	196.00
	Positive ranks	22n	16.59	365.00
	Ties	1o		
	Total	34		
TDTA post-pandemic - TDTA pre-pandemic	Negative ranks	8p	11.00	88.00
	Positive ranks	12q	10.17	122.00
	Ties	14r		
	Total	34		

a. ROA post-pandemic < ROA pre-pandemic.

b. ROA post-pandemic > ROA pre-pandemic.

c. ROA post-pandemic = ROA pre-pandemic.

d. EPS post-pandemic < EPS pre-pandemic.

e. EPS post-pandemic > EPS pre-pandemic.

f. EPS post-pandemic = EPS pre-pandemic.

g. ROE post-pandemic < ROE pre-pandemic.

h. ROE post-pandemic > ROE pre-pandemic.

i. ROE post-pandemic = ROE pre-pandemic.

j. TAT post-pandemic < TAT pre-pandemic.

k. TAT post-pandemic > TAT pre-pandemic.

l. TAT post-pandemic = TAT pre-pandemic.

m. DER post-pandemic < DER pre-pandemic.

n. DER post-pandemic > DER pre-pandemic.

o. DER post-pandemic = DER pre-pandemic.

p. TDTA post-pandemic < TDTA pre-pandemic.

q. TDTA post-pandemic > TDTA pre-pandemic.

r. TDTA post-pandemic = TDTA pre-pandemic.

**Table 4:** Test statistics.

	**ROA post-pandemic - ROA pre-pandemic**	**EPS post-pandemic - EPS pre-pandemic**	**ROE post-pandemic - ROE pre-pandemic**	**TAT post-pandemic - TAT pre-pandemic**	**DER post-pandemic - DER pre-pandemic**	**TDTA post-pandemic - TDTA pre-pandemic**
Z	−1.581b	−2.599c	−0.011b	−1.957b	−1.510c	−0.644c
Asymp. Sig. (2-tailed)	0.114	0.009	0.991	0.050	0.131	0.519

a. Wilcoxon signed ranks test.

b. Based on positive ranks.

c. Based on negative ranks.

Table 5 presents the descriptive statistics of the banks listed on the South Asian markets untill December 2021. The average returns of all banks in south Asian markets were negative during the pandemic period. However, because the COVID-19 epidemic in Pakistan is less severe than in other nations, the average return increases year after year. The data also suggests that the average return in Sri Lanka is lower since the country is more influenced by COVID-19. As a result, the country's financial performance suffers more during a pandemic. This result is consistent with Goodell (60) study that the financial sector is vulnerable during a pandemic and economic recession because of the possible occurrence of excessive bad loans and massive deposit withdrawals in a short time. The overall results show that all the south Asian Bankss' performance was positively influenced by the lockdown. Finally, our empirical findings back up (60) study, which focuses on the harmful consequences of COVID-19 on the banking industry. Because of the high risk of increasing bad debts and abnormally large withdrawals that may cause corporate crises or even bankruptcy, financial firms' stocks are among the most seriously affected securities on stock markets during a pandemic.

**Table 5:** Year wise comparison of financial ratios.

	**Pakistan**		**Sri Lanka**		**Bangladesh**	
	**Pre-pandemic**	**Post-pandemic**	**Pre-pandemic**	**Post-pandemic**	**Pre-pandemic**	**Post-pandemic**
ROA	0.67	0.62	0.96	0.83	0.57	0.15
ROE	0.04	0.19	0.10	0.08	0.10	0.06
EPS	6.36	9.11	11.28	10.02	2.21	1.76
TATR	3.77	3.98	10.23	9.30	6.88	5.79
DER	13.84	14.50	9.61	8.99	9.94	9.71
TDTA	0.91	0.94	0.90	0.91	1.36	1.06

### 4.5. Endogeneity

The results of Table 6 endogeneity show that the model has endogeneity because the probability of Durbin and Wu-Hausman F (1,312) is 0.7054, which is insignificant and accepts the null hypothesis that variables are exogenous. There is no evidence of endogeneity in the model.

**Table 6:** The result of endogeneity.

Durbin (score) chi2 (1) = 0.146799	(*p* = 0.7016)
Wu-Hausman F (1,312) = 0.143195	(*p* = 0.7054)

Table 7 shows the average return in terms of net profit of the south Asian banking industry. It's clearly shows that the results of the net profitability are decrease during the 2019 and 2021. But the net profitability of the banks is sufficient in 2016 to 2018. And the main reasons of decreasing the profitability during 2019 to 2021 is the COVID-19 pandemic affects. Because of the businesses are close down during pandemic. But banking fall in the services sector so, they don't stop their operations completely. They working during the period of pandemic through different modes like internet banking or through internet apps.

**Table 7:** Year wise average return.

	**2016**	**2017**	**2018**	**2019**	**2020**	**2021**
Pakistan	9,387,866	7,349,904	8,115,707	9,711,998	13,387,714	15,569,404
Sri Lanka	204,113,303	260,043,389	277,435,309	278,363,518	148,781,698	196,162,419
Bangladesh	2,657,230,520	2,709,283,311	2,666,835,936	2,498,227,830	2,300,527,704	1,992,160,397
Total	2,870,731,688	2,976,676,604	2,952,386,952	2,786,303,345	2,462,697,116	2,203,892,220

## 5. Discussion

The goal of this study was to assess the financial performance of the banking industry before and after the epidemic. The COVID-19 has a great impact on each business, especially the south Asian banking sector. During the pandemic period, Pakistan's government is also fighting against COVID-19 through social distancing and lockdown (61). The National Bank of Pakistan continues its operations through advanced technology (internet). Mostly, banks have shifted to a mobile app, and a user can make any transaction (withdraw, transfer) by using this app. Same as that in Bangladesh, where the government declared a general holiday for 2 months from March 26 to May 30 (62). The banks had continued their work on a small scale during the pandemic situation (63). They are also using the latest technology and providing e-services to their customers. Then there is no need to attack the branches physically. This will also maintain the social distance because no one will come to the branches. Sri Lanka, another famous tourist destination in the South Asian region, is also affected by this pandemic. The economy of said country is also dependent on the services sector, and the economy is declining badly. The country removes travel restrictions in 2021, which is a positive sign of recovery of 0.1% in said year (64). In Sri Lanka, the banks are also continuing their banking operations during COVID-19 for many reasons. One of the most important reasons for continuous operation in Sri Lanka is that the banks are responsible for payment and settlement systems. Therefore, the central bank of Sri Lanka (CBSL) authorizes the banks to regulate and supervise the payment, clearing, and settlement systems. Hence, banking operations continue during the period of COVID-19 in the south Asian banking sector. These banks perform their operations through the OTS (online transaction system), and some banks have shifted to mobile apps through which a customer can get the same services as banks. In the period, those banks have decreased their margins because they are not using the internet or advancing in technology. Therefore, in 2021, banks will improve their financial performance. In South Asia, the banking sector works for the government in the collection of taxes and the disbursement of cash for government expenditures. And almost all of the businesses working around the globe also depend on the banking sector. If the banks are closed during COVID-19, then the most critical situations will happen, and every business will face them. The banks play a vital role in emerging economies; if they close their operations during the pandemic period, the economies will face the following problems: lack of short-term and long-term capital financing; increased unemployment; a falling economic growth rate; increased interest rates; and much more (65).

The COVID-19 has negative impact on the financial performance of the banking industry in South Asia. As a result, the ROA of banks' assets has decreased throughout the epidemic era. As a consequence, the first hypothesis is accepted that there is significant difference in return on assets pre and post pandemic of south. The return on assets more decrease during the period of COVID-19. This finding supports the recent research of (63, 65), who discovered that the ROA decreased during the COVID-19 period. The second theory is concerning EPS, which is accepted and confirmed by Aprilia and Oetomo (66). Based on the rank test, the third hypothesis demonstrated that there is a difference in ROE between the pre and post pandemic periods, and the result is consistent with the previous study by Esomar and Christianty (67), in which the author stated that there were significant differences in ROE before and during the COVID-19 pandemic. The alternative explanation is also acceptable, since statistical data demonstrates a difference in performance between the pre and post pandemic periods. Daryanto and Rizki (58) prior investigation yielded similar results.

Finally, the banking sector plays a very important role in the economic development and growth of a country (68). But the overall performance of the banking sector is affected by many factors. COVID-19 is considered an important factor that affects the financial performance of the banking sector in South Asia. In this situation, the banking sector improves its performance through many indicators, which include IT adoption, technological advancement, and improved customer experience. A study by Dadoukis et al. (69) confirms that high IT-adopters improve their performance as compared to low IT-adopters in the period of the pandemic. All the businesses are closed during this time, but the banks are using the latest technology to run the business smoothly. Customers can perform transactions from their homes without standing in line at the banks. During the period of the pandemic, most banks will waive their transaction charges (free bank transfers through apps). The advancement in technology is measured through different things like mobile apps, social factors, free e-transactions, etc. This study is in line with previous research (70), which states that technology advancement is very influential in employee performance, ultimately increasing the performance of South Asian banks. The results of this study will also aid policymakers in developing policies to handle such crucial situations.

## 6. Conclusion

The purpose of this study is to analyse the financial performance of the South Asian banking sector before and after the COVID-19 pandemic. Using overall performance measures, liquidity, solvency, profitability, and activity ratios, it is found that the company had better performance before the pandemic. But the same was disturbed during the period of the COVID-19 pandemic, resulting in a decline. Another finding that we made was that the performance before and after the pandemic had significant differences, mainly in liquidity ratios, solvency ratios, and profitability ratios. Finally, we can conclude that banks can maintain their position in the business world because they do not stop working even in the pandemic period. They offer their service through technological advancement, and in the period 2021, they improve their performance as compared to the other pandemic periods. The said study is helpful for business owners, shareholders, and governments to understand the effect of COVID-19 on financial performance, especially in the banking sector, which has greatly contributed to the development of the country. We exclusively compared the organizational performance of the South Asian banking sector before and after the COVID-19 period. We gathered data from the most recent years, from 2016 to 2021, to assess organizational effectiveness. There are numerous other factors that influence stock markets during pandemics; however, this study did not take these into account and instead focused solely on the COVID-19 pandemic. Meanwhile, this study only examined the banking sector; further research might be conducted to examine other manufacturing sectors in order to provide more generalized conclusions. Furthermore, the study can be used to assess the organization's performance in relation to other variables, such as management strategies and decisions taken during the pandemic.

## Data availability statement

The raw data supporting the conclusions of this article will be made available by the authors, without undue reservation.

## Author contributions

ZM, SUQ, and ML contributed to conception and design of the study. SQ organized the database. SUQ and MR performed the statistical analysis. CY, ML, and SUQ wrote the first draft of the manuscript. ZM, SUQ, SQ, and MR wrote sections of the manuscript. HX contributed to correlation matrix and interpret results. All authors contributed to the article and approved the submitted version.
